# Sodium selenate regulates the brain ionome in a transgenic mouse model of Alzheimer’s disease

**DOI:** 10.1038/srep39290

**Published:** 2016-12-23

**Authors:** Lin Zheng, Hua-Zhang Zhu, Bing-Tao Wang, Qiong-Hui Zhao, Xiu-Bo Du, Yi Zheng, Liang Jiang, Jia-Zuan Ni, Yan Zhang, Qiong Liu

**Affiliations:** 1 College of Life Sciences & Oceanography, Shenzhen University, Shenzhen, 518060, Guangdong Province, P. R. China; 2 Shenzhen Entry-exit Inspection and Quarantine Bureau, Futian Huanggang Port, Shenzhen, 518033, Guangdong Province, P. R. China; 3 Shanghai Synchrotron Radiation Facility (SSRF), Shanghai Institute of Applied Physics, Chinese Academy of Sciences, Shanghai 201204, P. R. China; 4 Key Laboratory of Nutrition and Metabolism, Institute for Nutritional Sciences, Shanghai Institutes for Biological Sciences, Chinese Academy of Sciences, University of Chinese Academy of Sciences, Shanghai 200031, P. R. China

## Abstract

Many studies have shown that imbalance of mineral metabolism may play an important role in Alzheimer’s disease (AD) progression. It was recently reported that selenium could reverse memory deficits in AD mouse model. We carried out multi-time-point ionome analysis to investigate the interactions among 15 elements in the brain by using a triple-transgenic mouse model of AD with/without high-dose sodium selenate supplementation. Except selenium, the majority of significantly changed elements showed a reduced level after 6-month selenate supplementation, especially iron whose levels were completely reversed to normal state at almost all examined time points. We then built the elemental correlation network for each time point. Significant and specific elemental correlations and correlation changes were identified, implying a highly complex and dynamic crosstalk between selenium and other elements during long-term supplementation with selenate. Finally, we measured the activities of two important anti-oxidative selenoenzymes, glutathione peroxidase and thioredoxin reductase, and found that they were remarkably increased in the cerebrum of selenate-treated mice, suggesting that selenoenzyme-mediated protection against oxidative stress might also be involved in the therapeutic effect of selenate in AD. Overall, this study should contribute to our understanding of the mechanism related to the potential use of selenate in AD treatment.

Alzheimer’s disease (AD) is a progressive and fatal brain disease coupled with the decline of cognitive ability and loss of memory. It is characterized by the presence of amyloid-β (Aβ, mainly Aβ_40_ and Aβ_42_)-containing plaques (APs) and hyperphosphorylated tau-containing neurofibrillary tangles (NFTs) in pathological brain tissue^1^,^2^. APs are extracellular deposits of Aβ in the grey matter of the brain and have been considered to be neurotoxic^3^, whereas the tau protein is a microtubule-binding protein whose main function is to maintain axonal microtubule stability. To date, the underlying mechanism linking Aβ and tau to AD still keeps uncertain^1^,^4^,^5^. Many testing drugs have been designed to target established mechanisms of AD, especially the removal of aggregated Aβ^6^,^7^. Unfortunately, almost all of them have failed to benefit patients. Thus, new effective treatment strategy is urgently needed.

Recent studies have shown that AP and NFT pathologies may not be the only reason for AD development^8^. Some other factors, such as metal dyshomeostasis^9^, mitochondrial function impairment^10^ and insulin resistance^11^, are also involved in the pathogenesis of AD. Metals are essential for maintaining normal physiological function of all organisms. Metal dyshomeostasis is frequently observed in AD patients due to anomalous binding of metals such as iron (Fe), copper (Cu) and zinc (Zn), or impaired regulation of redox-active metals which can induce the formation of reactive oxygen species (ROS) and neuronal damage^12^,^13^,^14^,^15^,^16^. Targeting some of these metals might be an alternative approach to treat this disease^17^. For example, metal chelation has been reported to be used in AD transgenic mice and clinical trials, which may become a potential therapy for AD^18^,^19^. Considering that AD represents a complex neurological disorder which involves the variation of multiple metals and minerals^20^,^21^,^22^,^23^, studies based on individual elements could not provide a comprehensive view of metal homeostasis disruption in AD. In recent years, the terms metallome (the complete set of metal ions in an organism) and its extension ionome (all mineral nutrients and trace elements found in an organism) have been introduced^24^. The study of the ionome, ionomics, involves quantitative high-throughput profiling of elemental composition in living systems, usually by inductively coupled plasma-mass spectrometry (ICP-MS)^25^,^26^,^27^. Now ionomics is emerging as an important tool for increasing our understanding of ionome homeostasis in various physiological and pathological conditions^25^,^28^. Unfortunately, a systematic view of the ionome in either AD mouse models or patients is still lacking.

Additionally, selenium (Se), which is an important trace element and plays a critical role in various redox and metabolic processes, has been suggested to be used in the prevention of the onset and progression of AD^29^,^30^. For example, researchers found that high-dose dietary supplementation of selenite could reduce the amount of senile APs in the brain using Tg2576 transgenic mice^31^. Recently, another Se form, selenate, has also aroused researchers’ interest due to its lower physical toxicity and outstanding performance in reducing tau hyperphosphorylation, which may become a promising novel therapeutic drug in AD^32^,^33^,^34^. Se is mainly used in the form of selenocysteine which is found in the active sites of selenoproteins (e.g., human has 25 selenoproteins^35^). Thus, many of the protective effects of Se are thought to be mediated by selenoproteins, especially key selenoenzymes involved in antioxidant defense and redox regulation, such as glutathione peroxidases (GSH-Pxes) and thioredoxin reductases (TrxRs)^36^,^37^. On the other hand, as the major metalloid micronutrient, the interaction between Se and other metals or minerals is not clear yet.

In this study, we tried to investigate the effect of selenate in the treatment of AD and its impact on some other elements in the brain. A significant number of triple-transgenic mice of AD (3×Tg-AD, harboring mutations of Psen1^tm1Mpm^, APPSwe, and tauP301L transgenes; APs and NTFs could be found in both cortex and hippocampus at 6–8 months of age) were fed with sodium-selenate-enriched diet. We analyzed the ionome profiles in the cerebrum of Se-treated 3×Tg-AD mice, non-Se-treated 3×Tg-AD mice as well as wild-type (WT) mice at multiple time points using ICP-MS techniques. Differentially changed elements (DCEs) were identified between different groups at each time point. We further explored the relationship between different elements based on correlation network analysis. To our knowledge, this is the first effort to study the ionome patterns in mouse brain that may help understand the role of selenate in the treatment of AD.

## Results

### High-dose sodium selenate supplementation alleviates AD pathology in 3×Tg-AD mice

**Figure 1:**
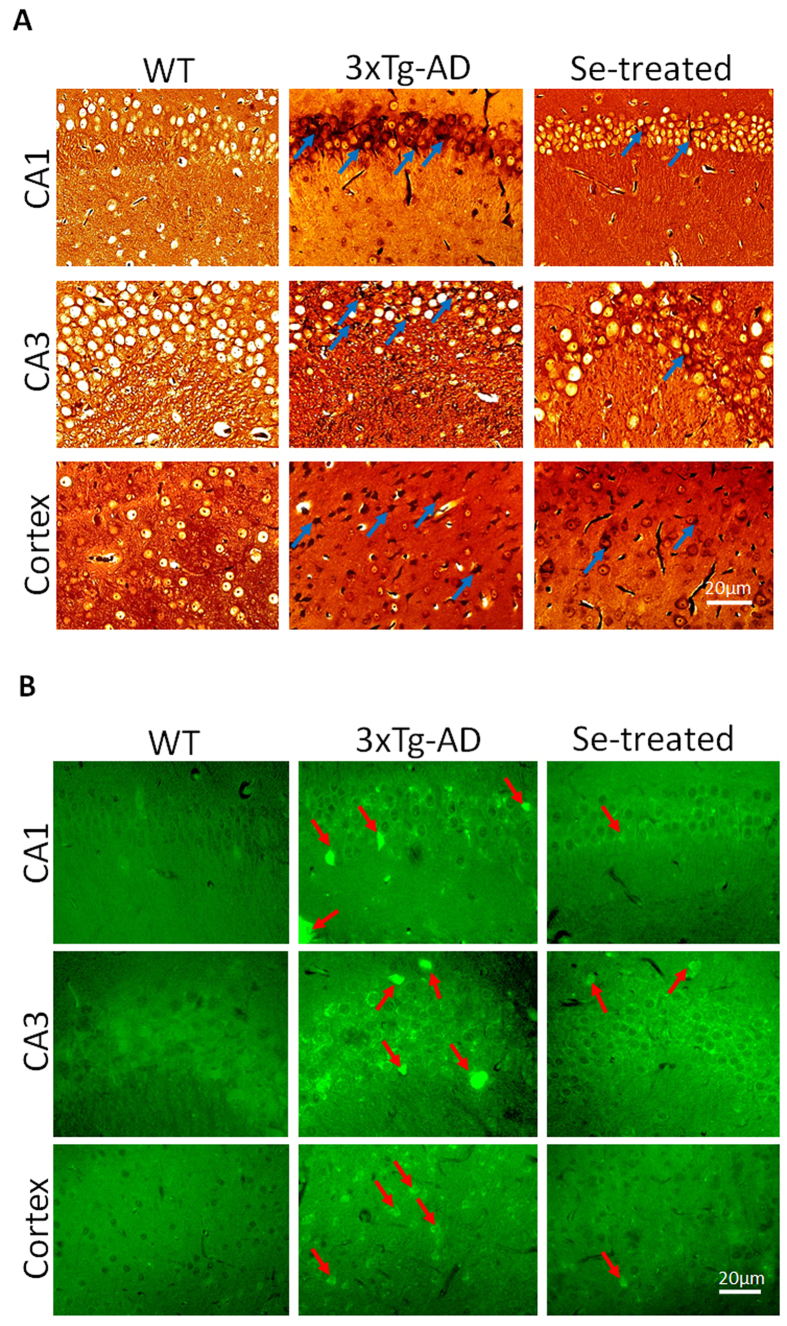
AD-related pathological features in the brains of different groups of mice. (**A**) The Gallyas-Braak silver staining of NFTs in the brains of 12-month-old mice: a significant reduction of NFTs was observed in the Se-treated group compared with the 3×Tg-AD group (indicated by blue arrows); (**B**) thioflavin S staining (40×) of APs in the brains of 12-month-old mice: In the Se-treated group, the amount of stained APs was decreased, and they were relatively uniform and decentralized compared with those in the 3×Tg-AD group (indicated by red arrows).

After 10-month’s sodium selenate supplementation (starting from the age of 2 months), mice in the Se-treated 3×Tg-AD (named Se-treated thereafter) group showed a significant relief of AD pathology when compared with the non-Se-treated 3×Tg-AD (named 3×Tg-AD thereafter) group. For example, NFTs were observed to be significantly increased in the hippocampi and cortices of 3×Tg-AD mice (compared with WT) at 12 months old using Gallyas-Braak silver staining, whereas they were largely reduced in the same regions of Se-treated 3×Tg-AD mice (Fig. 1A). Similarly, thioflavin S staining showed that Aβ aggregates were significantly increased in the hippocampi and cortices of 3×Tg-AD mice; however, selenate supplementation could significantly inhibit their formation in different degrees (Fig. 1B).

### General analysis of the ionome in transgenic mouse brain

**Figure 2:**
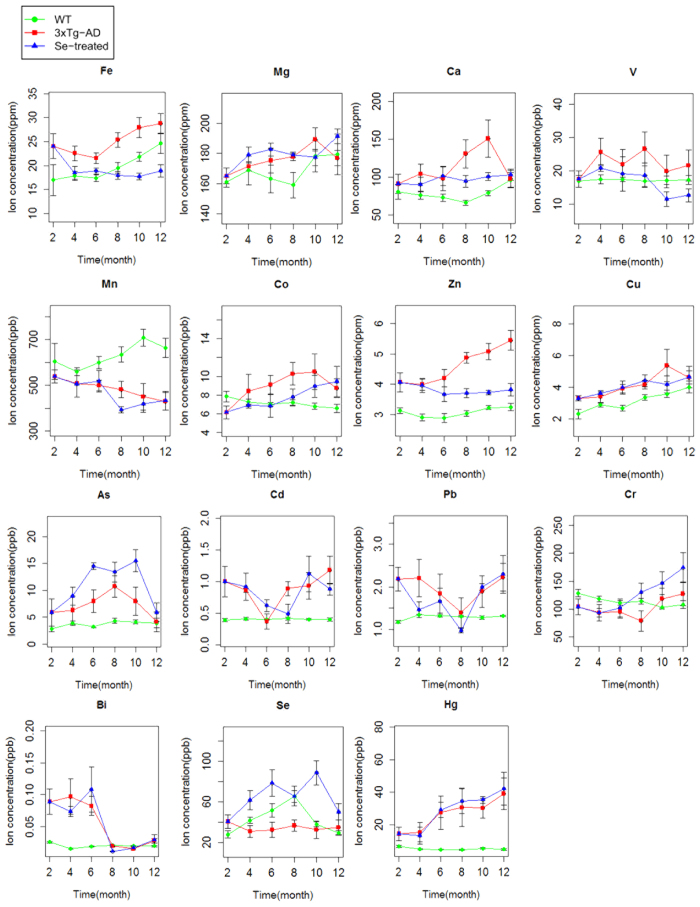
Distribution of elements across groups and time points. Green, red and blue lines represent the WT, 3×Tg-AD and Se-treated groups, respectively. Bars represent mean values ± 95% confidence interval (n = 6).

We measured the concentrations of 15 elements (mostly metals) in the brain samples of Se-treated, 3×Tg-AD and WT mice. Details are shown in Fig. 2.

First, we compared the 3×Tg-AD with WT groups. Almost all examined elements (12 out of 15) were considered as DCEs at one or more time points (p < 0.05, Table 1), which was also supported by repeated-measures analysis of variance (ANOVA). The majority of them appeared to be significantly enriched in the brain tissues of 3×Tg-AD mice for a relatively long period, including Fe, Zn, Cu, cadmium (Cd), mercury (Hg) and bismuth (Bi) whose levels in the brain have been reported to be elevated in AD^38^,^39^,^40^,^41^,^42^,^43^,^44^,^45^,^46^. Excess Fe has been linked to the development of AD, which may combine with Aβ in the APs to accelerate neuronal Aβ production and cognitive impairment^39^. Zn is an essential trace element for the brain development and its proper functions, whose misbalance is thought to be involved in the pathogenesis of AD^40^. In addition, aberrant Cu metabolism is also a potential risk factor accelerating AD^41^. Higher Hg concentrations were found in brain regions and blood of some patients with AD^42^. Accumulation of Cd and Bi in the brain or serum was observed to be significantly associated with AD^43^,^44^,^45^,^46^. Some other elements with increased levels in the brain of AD mice at certain time points (including arsenic (As) and lead (Pb)) are also known as neurotoxic factors. In contrast, levels of very few elements, such as manganese (Mn) and Se, were found to be reduced in 3×Tg-AD mice. The close correlation between Se deficiency and the risk of AD has been shown in previous studies^30^. However, Mn appeared to have an opposite trend from that reported previously: overexposure to Mn might be involved in the progression of AD as well as other neurodegenerative diseases^12^,^47^,^48^,^49^. Further studies are needed to investigate the relationship between Mn and AD progression. In general, our data agreed well with previous observations.

Second, we analyzed DCEs between Se-treated and 3×Tg-AD mice. A total of ten DCEs were identified (mostly consistent with repeated-measures ANOVA analysis), with an overlap of 7 elements when compared with the above AD-associated elements (Table 1). This finding revealed an important crosslink between Se and the homeostasis of other elements. The levels of almost all DCEs were decreased, especially after 6-month selenate supplementation. The largest number of DCEs (seven elements) was observed at the age of 8 months. As expected, Se level has been consistently elevated from the early stage (4 months old), most likely due to high-dose selenate intake. Interestingly, concentrations of Fe and Zn appeared to be significantly reduced since 8 months old, suggesting that depleted levels of these two AD-related metals may contribute to the role of selenate in the treatment of AD. On the other hand, the concentration of Cu was not significantly changed with selenate intake.

Three other selenate-induced DCEs (magnesium (Mg), vanadium (V) and chromium (Cr)) are known to be essential for mammals; however, their levels were not significantly changed between 3×Tg-AD and WT mouse groups. It was reported that Mg has neuroprotective effect, whose deficit in the serum or brain might be a risk factor for AD^50^,^51^,^52^,^53^. To date, the relationship between V or Cr and AD is not clear. It has been reported that Cr is related to progressive dementias and oxidative stress^54^,^55^.

The remaining four DCEs include Mn, Cd, Bi and As. Since Mn, Cd and Bi might be risk factors for AD (see above), decreasing their levels at certain time points might be helpful in the treatment of AD. As is a neurotoxic metalloid with adverse effects in both neurodevelopment and cognitive function, whose exposure is also considered as a risk factor for AD development^56^. Here, elevated levels of As were observed at the age of both 6 and 10 months in the Se-treated group. Although the mechanism is not clear, it was suggested that the use of adequate Se could become a therapeutic strategy to combat As toxicity^57^. Overall, our results suggest that high-dose selenate supplementation could significantly influence the ionome in the brain and, in consequence, reduce the risk of AD.

### Clustering analysis of ionomes

ICP-MS-based ionomic analysis provided us with an opportunity to identify possible functional linkages among elements by comparing their patterns. To this end, we subjected the ionome dataset to clustering analysis using R package. The ionome profiles were z-score transformed and then analyzed by hierarchical clustering approach. The correlation heatmaps from different groups of mice were similar (Supplementary Fig. S1), suggesting that the functional associations between these elements were generally conserved. A few clusters of elements with similar distribution patterns were observed. In all groups, Zn and Cu, Fe and V, as well as Pb, Cd and Bi were always clustered together whereas Mn was always an outgroup. In addition, difference could be observed for each group. For example, strong correlation between Se and calcium (Ca) was only observed in Se-treated mice, implying that Se supplementation might be associated with Ca metabolism which is very important for maintaining the proper function of neuron.

We also used different classification algorithms to separate Se-treated and 3×Tg-AD groups regardless of time points. Several methods (such as naive Bayesian classifier, decision tree analysis, random forest, etc.) were used. Among them, the decision tree algorithm gave the best performance, whose accuracy rate is 92% (only based on two elements: Fe and Se, Supplementary Fig. S2). These results suggest that Fe content could be used as a potential marker for evaluating the therapeutic effects of selenate supplementation in the transgenic mouse model of AD.

### Elemental correlation analysis

**Figure 3:**
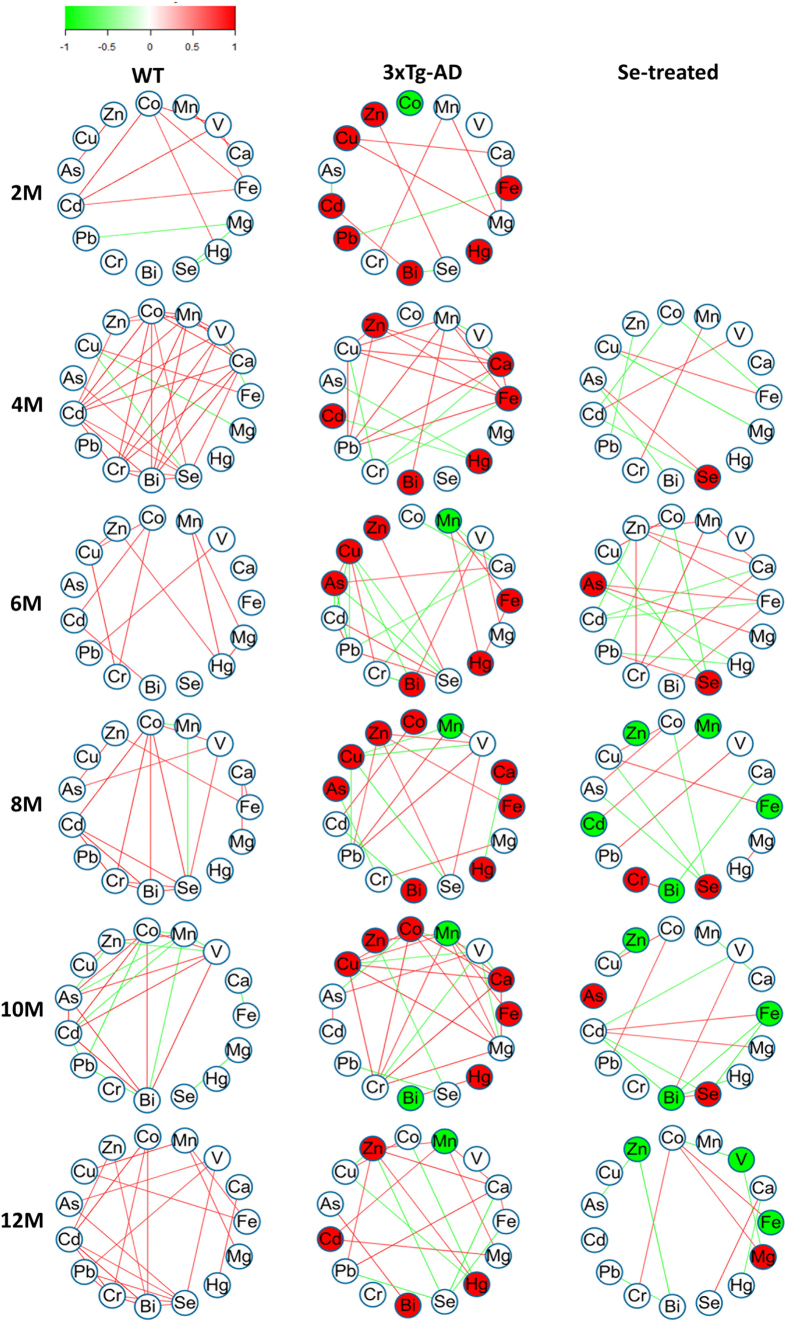
The elemental correlation network. Only significant pairwise SCCs whose values are greater than the threshold are shown. Nodes represent different elements. DCEs are highlighted in red (increased) and green (decreased). Green and red lines represent negative and positive correlation, respectively. Different color scales represent the magnitude of SCCs.

Elemental correlation analysis has been used in several ionome-based studies^58^,^59^. To further explore the relationship between elements examined in mouse brain, we calculated the Spearman correlation coefficients (SCCs) for all possible element pairs (105 pairs in total; Supplementary Table S1), and built the elemental correlation network for each group at each time point (Supplementary Fig. S3). A condensed version of the network which contains significant SCCs (SCC > 0.749 as defined in Materials and Methods) is shown in Fig. 3.

Almost all element pairs were found to be significantly correlated at one or more time points in at least one group, suggesting a complex relationship between these elements. Significant correlations were highly variable, which depended on different time points and groups. Only Fe and Cu correlated positively at the age of 4 months in all three groups, which is consistent with previous findings that Cu is essential for Fe metabolism^60^. In general, much fewer significant SCCs were identified in the Se-treated group when compared with 3×Tg-AD and WT groups, implying that selenate supplementation could weaken the interactions among different elements (Supplementary Table S1). In addition, a small number of significant correlations were exclusively present in certain groups (mostly at single time points), especially those in either 3×Tg-AD or Se-treated groups (Table 2). For example, strong positive correlations between Se and Zn and between Ca and Cu were found in the 3×Tg-AD group but were absent in both Se-treated and WT groups, suggesting that these elemental interactions might be involved in AD progression and could be eliminated by selenate intake. Our data reveal clearly that, besides altering the levels of elements, selenate supplementation could influence element-element crosslinks in the brain even if such effect is time-dependent.

It should also be noted that, although the majority of SCCs were not significant at each time point, difference of SCCs between different groups (|ΔSCC|) might be significant (or named differentially changed correlation, DCC). Similar strategies have been applied to investigate new mechanisms in complex diseases^61^,^62^. Here, we identified 63 and 49 DCCs between 3×Tg-AD and WT groups (or named AD-related DCCs) and between Se-treated and 3×Tg-AD groups (or named Se-related DCCs), respectively, with an overlap of 30 DCCs (Supplementary Table S2). Surprisingly, opposite trends were observed for most of the overlapped DCCs, especially at the early stage after selenate supplementation. For example, at the age of 4 months, ΔSCC of Fe-Mn between 3×Tg-AD (SCC: 0.886) and WT (SCC: –0.6) groups was 1.486, whereas it became –1.205 between Se-treated (SCC: –0.319) and 3×Tg-AD groups, implying that selenate could significantly reverse the correlation between Fe and Mn altered in AD mice and bring it back close to the normal state in WT mice. Therefore, our results suggest that selante supplementation could significantly affect the interactions among different elements at two levels: (i) elemental correlation; (ii) changes of such correlation, both of which might contribute to the therapeutic effects of selenate in AD transgenic mice.

### Analysis of key selenoenzymes’ activity in mouse brain

To further investigate the regulatory effect of selenate intake on selenoproteins in the brain, especially for key anti-oxidative selenoenzymes, we analyzed the activities of two of them: GSH-Px and TrxR. GSH-Px is an important peroxide-decomposing enzyme whose level could reflect the anti-oxidative capacity of mice and the bioavailability of Se. TrxR may reduce thioredoxin to keep redox balance *in vivo*. Those two selenoenzymes play important roles in Se metabolism and protection against oxidative stress^35^,^36^,^37^.

**Figure 4:**
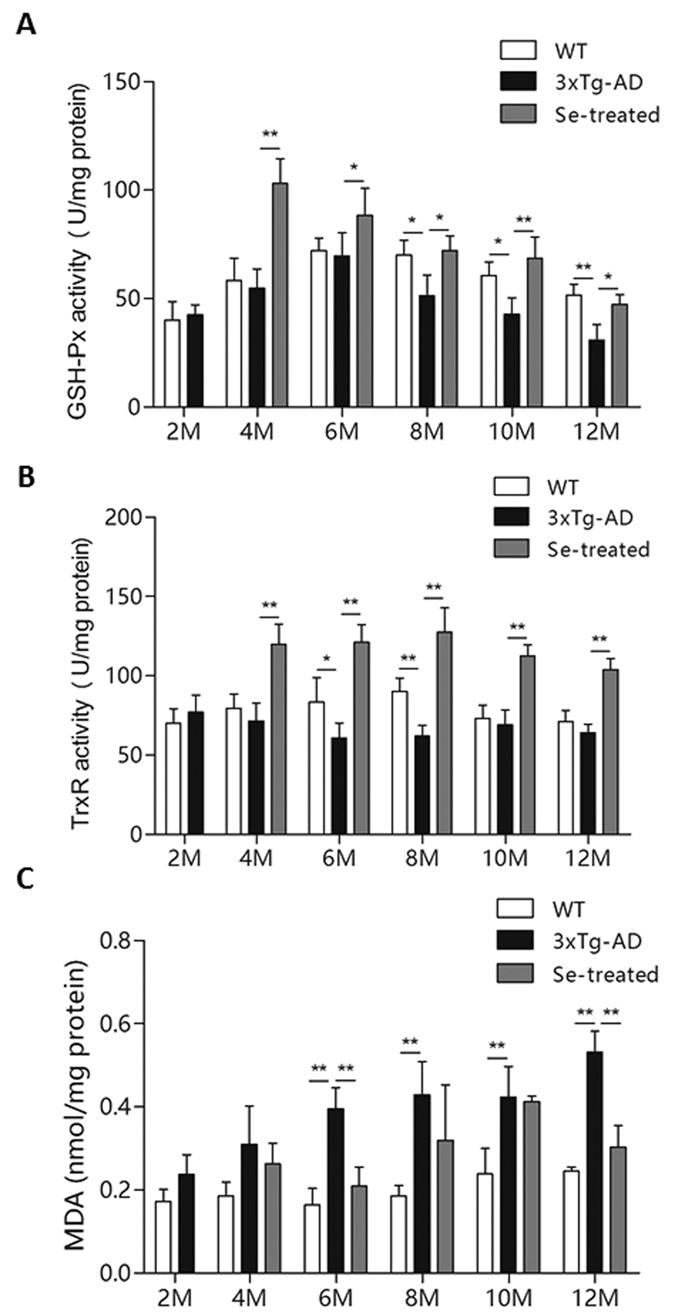
Measurements of the activities of selenoenzymes and MDA levels in the brains of different groups of mice. (**A**) GSH-Px activity; (**B**) TrxR activity; (**C**) MDA level (n = 6; **p < 0.01).

Here, we observed significantly increased activities of GSH-Px and TrxR in the cerebral tissues of 3×Tg-AD mice with selenate supplementation (Fig. 4). Such an effect happened from the early stage (4 months old) and lasted until late stage (12 months old). The enhancement of GSH-Px activity was observed to reach a peak level after two months of selenate intake (4 months old), and then decreased gradually with age (Fig. 4A). On the other hand, the TrxR activity appeared to be relatively stable after a significant increase at 4 months old (Fig. 4B). Accordingly, the levels of malondialdehyde (MDA) were significantly decreased at the age of 6 and 12 months old after selenate supplementation (Fig. 4C). Thus, our results suggest that selenate could also inhibit lipid peroxidation and oxidative damage occurred in the brains of AD mice through the increased activities of GSH-Px and TrxR.

## Discussion

Both essential micronutrients (such as Zn, Fe, Cu and Se) and other minerals are thought to correlate with the progression of many neurodegenerative diseases such as AD. Disproportion of these elements, especially heavy metal accumulation in the brain, may cause severe damage to the central nervous system and finally lead to neurodegeneration and cell death^38^. In spite that some metal chelators (say, Fe chelators) could decrease the concentration of certain metals to some extent in the brains of AD mouse models or patients, they may interfere with the normal metabolism of other metals and disrupt the whole mineral metabolic network. Therefore, it is a tremendous challenge to decrease the accumulation of toxic metals without changing the normal function of minerals in the brain and to improve the learning and memory impairments of AD patients.

In recent years, it has been shown that sodium selenate could improve contextual memory and motor performance of AD patients while preventing neurodegeneration. Several studies suggested that selenate may reduce tau phosphorylation both *in vitro* and *in vivo*, and mitigate the tau pathology via the activation of the serine/threonine-specific protein phosphatase 2A (PP2A)^32^,^63^, however, the underlying mechanism is still unclear. Supplementation with selenate has been reported to significantly increase Se level in blood and several tissues/organs (including brain) in both rodents and large mammals^64^,^65^. The relationship between Se content in plasma and that in the brain is very complex, which depends on a variety of factors such as blood-brain barrier and chemical forms of Se^66^. Previous analyses of plasma Se status in AD patients showed a contradictory relationship between Se levels and AD^67^,^68^,^69^, implying that plasma Se may not be fit for monitoring the effect of selenate in the treatment of AD. On the other hand, it has been known that Se-containing amino acids could chelate redox-active metal ions in different tissues^70^, which may somewhat affect metal utilization in the brain. Therefore, it should be very important to investigate the Se content in the brain and its relationship with other elements (especially AD-related metals) which is completely unknown.

In this study, we carried out an extensive time series analysis of 15 elements in the brains of Se-treated 3×Tg-AD mice, non-Se-treated 3×Tg-AD mice and WT mice. We were able to offer new insights on AD-induced changes of elemental composition of mouse brain, identify patterns of element utilization, and investigate the relationship between selenate and other elements. First, 12 DCEs were identified between 3×Tg-AD mice and WT mice, most of which are known to be related to AD onset and progression (such as Fe, Zn, Cu, Cd and Se), implying that the 3×Tg mouse is a good model for ionome study of AD. After long-term supplementation with selenate, 10 DCEs (including Se) were identified at one or more time points, especially Fe and Zn whose levels were significantly and persistently decreased after 6-month selenate supplementation. Neuronal Fe deposition in AD brains is a consistently reported finding in many studies, which may cause multiple types of damage such as increased Aβ production, elevated oxidative stress and even cell death^71^,^72^,^73^. Similarly, Zn could not only combine with Aβ to initiate plaque formation but also impact on tau-related neurotoxicity in AD^17^. Besides, levels of some other AD-related elements (such as Cd and Bi) were also found to be significantly decreased at certain time points. To further clarify whether levels of some of these elements could be reversed to their normal ranges, we compared the element concentrations between Se-treated and WT mice (Supplementary Table S3). Although all 15 elements were considered as DCEs at certain ages, only the Fe level was observed to return to normal or even lower state at all examined time points, whereas the Zn level was decreased somewhat but still higher than its normal range at all time points. Thus, it appears that restoration of Fe level in the brain may play a key role in therapeutic effects of selenate in transgenic AD mice.

Clustering analysis revealed that several element clusters were same among different groups, suggesting that they might be regulated using similar strategies. Interestingly, in the Se-treated group, Se was found to be closely correlated with Ca whose homeostasis and signaling are essential for proper neuron function. Although the Ca level was not significantly changed after selenate supplementation, a strong correlation between Se and Ca suggests that Ca metabolism might be involved in selenate-related AD prevention. In addition, decision tree-based classification analysis revealed that Fe could be a good marker for evaluating the effect of selenate for AD transgenic mice, which is consistent with the above idea that decreasing the Fe level might be a key factor for AD treatment using high-dose selenate. Although the mechanism how Se could affect Fe metabolism in the brain is still unclear, previous studies have shown that dietary Se can regulate expression of genes involved in Fe metabolism such as transferrin and its receptor genes^74^,^75^. Future research is needed to address this question.

To further investigate the impact of the interactions among these elements, we built the elemental correlation network which could offer valuable insights into the nature of the crosstalk in the brain ionome (including both DCEs and other elements). We identified all significant correlations at each time point for each group of mice, and found that some of them are group-specific. Moreover, we identified changed correlations (i.e., DCCs) across different groups. Surprisingly, selante supplementation could reverse a significant number of AD-related correlation changes and bring them back to the normal state in WT mice. Some of these reversed interactions are known to be essential for maintaining the normal brain function, such as Fe and Mn, Fe and Cu as well as Ca and Mn interactions^76^,^77^,^78^. For the first time, we showed that selenate supplementation may affect not only the levels of a number of elements in the brain, but the multiple associations and interactions among them as well. Compared with individual elements, dynamic and specific changes of the relationship between them could provide new clues about the complex interdependency between Se and other elements in both AD progression and treatment.

It has been suggested that Se supplementation is able to increase the anti-oxidative ability of cells. As the major biological use of Se is to synthesize selenoproteins, we examined the activities of two important selenoenzymes, GSH-Px and TrxR. The activities of both proteins were significantly increased from the early stage after high-dose selenate supplementation, which could also contribute to AD treatment using high-dose sodium selenate.

Finally, we proposed two hypotheses for the potential mechanisms of the effect of selenate: First, selenate is able to reduce the levels of certain elements that are known as AD risk factors (especially Fe), change the interactions among elements that might be involved in AD progression, and in turn inhibit the production and/or aggregation of Aβ; Second, the activities of several key anti-oxidative selenoenzymes could be significantly increased, which may help restore the normal function of neurons in the brain of AD mouse model. Further efforts are needed to investigate the mechanism of the complex interactions between Se and other elements to understand how Se could influence the metabolism and homeostasis of different minerals as well as their relationship with AD.

In conclusion, this is the first attempt to study the complex relationship between Se and other elements in the treatment of 3×Tg-AD mice using high-dose sodium selenate. Significant differences at three levels (individual elements, elemental correlations and changes of such correlations) were identified at each examined time point, which demonstrates a highly dynamic and somewhat specific effect on brain ionome induced by selenate supplementation. In addition, elevated activities of important selenoenzymes may also correlate to the role of selenate. Together, these findings provide direct insights into how selenate supplementation can adjust the brain ionome of AD mouse model, and help to understand the mechanism by which selenate could restore impaired memory in AD.

## Materials and Methods

### Ethics statement and source of experimental animals

The study was approved by the Institute of Marine Biotechnology at Shenzhen University, and performed in accordance with relevant guidelines and regulations. The parental mice were purchased from the Jackson laboratory and propagated in the College of Life Sciences and Oceanography at Shenzhen University.

### Animal preparation

Two 3×Tg-AD mouse groups (Se-treated and non-Se-treated 3×Tg-AD groups) and one WT group of male mice were used in this study. In the first two months after birth, all three groups were fed with the same amount of normal food (Se concentration was less than 0.1ppm; provided by Guangdong Medical Experimental Animal Center, Guangzhou, China). After that, 6 μg/mL sodium selenate (Sigma-Aldrich) dissolved in ultrapure water was added for the Se-treated group, the volume of water drunk by each mouse was 3–4 mL per day, which is equivalent to18–24 μg sodium selenate (7.5–10.0 μg Se) intake per day. Such a dose is much higher than what is recommended for daily intake; however, it is not high enough to cause Se toxicity^32^,^33^. All mice were kept in the same environment in the SPF Laboratory Animal Room.

### Brain sample processing

Mice of WT, 3×Tg-AD and Se-treated 3×Tg-AD were euthanized with ether anhydrous inhalation, and their brain tissues were rapidly removed from all mice in different groups at the ages of 2 (only for the WT and 3×Tg-AD mouse groups), 4, 6, 8, 10 and 12 months (six mice for each group at each time point, 102 mice in total). The cerebrum was separated into two hemispheres equally. The right hemisphere was further dissected into hippocampal and cortical samples, fixed with 4% paraformaldehyde for 24 h (Sinopharm Chemical Reagent, Shanghai, China), followed by dehydration with serial ethanol (Aladdin, Shanghai, China), clearing with xylene (Aladdin, Shanghai, China) and infiltration with paraffin. Those treated brain tissues were then embedded in high-purity paraffin (Leica) for the following experiments. The left hemisphere of the mouse cerebrum was accurately weighted, homogenated and divided into two parts: one part for the measurement of metal ions, another part for the measurements of selenoenzyme activity and MDA levels. All samples were stored at −80 °C before further analysis.

### Gallyas-Braak silver and Thioflavin S staining

The paraffin-embedded right hemisphere of the brain was cut into serial sections at 10 μm thickness on a freezing sledge microtome. Conventional Gallyas-Braak silver staining method was used to detect NFT generated from hyperphosphorylated tau inside cerebral neurons. Thioflavin S staining was performed to examine Aβ deposit outside cerebral neurons^79^,^80^.

### Element quantifications

One part of the homogenate of left hemisphere was digested with 10% GC nitric acid (Aladdin, Shanghai, China) in the Microwave Digestion System (CEM-Mars6) for 30 min at 150 °C. Digested liquid was collected with clean centrifuge tube (Corning, New York, U.S.A) and added with ultrapure water until 10 mL. All samples were analyzed for the elements of interest immediately. The concentrations of 15 elements including Se, Fe, Zn, Cu, Mg, Hg, Ca, Cr, V, Mn, Cd, cobalt (Co), Pb, As and Bi, were quantified using Agilent Technologies ICP-MS 7700x system (Agilent Technologies, Tokyo, Japan) equipped with an ASX-500 series autosampler at Shenzhen Entry-exit Inspection and Quarantine Bureau.

### Statistical analysis

Statistical analysis was performed using *R* language (version 3.1.1). For each element, individual outliers were identified using Dixon’s Q-test and then replaced with the average value of the rest samples at the same time point. Kolmogorov-Smirnov (KS) test^81^ was applied to determine whether the data fit the normal distribution. We found that the values of many examined elements did not come from a normal distribution. Thus, we used Wilcoxon test to identify DCEs between different groups if p-value < 0.05. *R*-based ANOVA with repeated measures was also used to analyze ionome data examined at different times. Hierarchical clustering and various classification methods (such as decision tree) were used for element clustering analysis and group classification, respectively.

### Elemental correlation network analysis

SCC was used to analyze the correlation between elements for different groups at each time point. Considering that correlation network analysis is a widely used systems biology approach for studying biological networks based on pairwise correlations between variables^82^, we further built the elemental correlation network to analyze the relationship between ionome and AD progression. In such a network, each edge represents the SCC value of the two corresponding elements (nodes). The threshold of significant SCC was determined via 1000-time random simulation with p-value < 0.05. The cutoff of 95% confidence interval was 0.749 ± 0.028. We used 0.749 as the threshold to identify significant SCCs.

In addition, we used a similar approach to identify DCCs at each time point if the difference of SCCs between two groups (|ΔSCC|) was greater than certain threshold. The cutoff of 95% confidence interval of |ΔSCC| was 1.050 ± 0.036. Thus, 1.05 was used as the threshold for DCCs (|ΔSCC| > 1.05). Finally, the correlation network was presented using Cytoscape software^83^.

### Selenoenzyme activity and malondialdehyde level measurements

The rest of the homogenate of left hemisphere was dissolved in RIPA Lysis Buffer with protease and phosphatase inhibitors and then centrifuged at 12000 rpm for 20 min at 4 °C. Soluble protein content of the homogenate was measured using the BCA Assay Reagent (Thermo). Two portions of 20 μg and 50 μg total proteins were used respectively for the measurement of GSH-Px and TrxR activities using the assay kits purchased from the Beyotime (Nantong, China) and Solarbio (Shanghai) companies, respectively. The level of MDA was measured on a UV-VIS spectrophotometer (Hitachi, Japan) at 535 nm and expressed as nmol/mg protein.

## Additional Information

**How to cite this article**: Zheng, L. *et al*. Sodium selenate regulates the brain ionome in a transgenic mouse model of Alzheimer’s disease. *Sci. Rep.* **6**, 39290; doi: 10.1038/srep39290 (2016).

**Publisher's note:** Springer Nature remains neutral with regard to jurisdictional claims in published maps and institutional affiliations.

## Supplementary Material

Supplementary Information

## Figures and Tables

**Table 1:** The list of DCEs between different mouse groups.

Age	DCEs (3×Tg-AD v.s. WT)		DCEs (Se-treated v.s. 3×Tg-AD)	
	Increased	Decreased	Increased	Decreased
2-month	Fe, Zn, Cu, Cd, Pb, Bi, Hg	Co	—	—
4-month	Fe, Ca, Zn, Cd, Bi, Hg	—	Se	—
6-month	Fe, Zn, Cu, As, Bi, Hg	Mn	Se, As	—
8-month	Fe, Ca, Co, Zn, Cu, As, Cd, Hg	Mn, Se	Se, Cr	Fe, Zn, Mn, Cd, Bi
10-month	Fe, Ca, Co, Zn, Cu, Hg	Mn, Bi	Se, As	Fe, Zn
12-month	Zn, Cd, Bi, Hg	Mn	Mg	Fe, Zn, V

**Table 2:** Significant and group-specific interactions among elements.

Group	Elemental interactions
WT	Se-Hg, Co-Zn, Mn-Se, Mn-As, Fe-V, Mg-Se, Co-Bi, Cd-Cr, Cr-Se
3×Tg-AD	Fe-Cr, Cd-Hg, Fe-Hg, Ca-As, Cu-As, Cu-Cd, Cu-Bi, As-Cr, Cu-Hg, Fe-Pb, Mg-Cr, Ca-Cu, V-Cu, Mn-Pb, Zn-Se, Cu-Pb
Se-treated	Fe-As, Mg-As, Zn-Cr, Pb-Hg, Fe-Mg, V-Hg, Fe-Bi
