# Supplementary material for: Sodium selenate regulates the brain ionome in a transgenic mouse model of Alzheimer’s disease
Source: Sci Rep. 2016 Dec 23;6:39290. doi: 10.1038/srep39290 (PMC5180247; doi:10.1038/srep39290)
Supplement: Supplementary Information [file srep39290-s1.pdf]

Sodium selenate regulates the brain ionome in a transgenic mouse model of Alzheimer's disease

Lin Zheng[1,†], Hua-Zhang Zhu[1,†], Bing-Tao Wang[2], Qiong-Hui Zhao[2], Xiu-Bo Du[1], Yi Zheng[3], Liang Jiang[1], Jia-Zuan Ni[1], Yan Zhang[1,4,*], Qiong Liu[1,*]

[1]: College of Life Sciences & Oceanography, Shenzhen University, Shenzhen, 518060, Guangdong Province, P. R. China

[2]: Shenzhen Entry-exit Inspection and Quarantine Bureau, Futian Huanggang Port, Shenzhen,518033,Guangdong Province, P. R. China

[3]: Shanghai Synchrotron Radiation Facility (SSRF), Shanghai Institute of Applied Physics, Chinese Academy of Sciences, Shanghai 201204, P. R. China

[4]: Key Laboratory of Nutrition and Metabolism, Institute for Nutritional Sciences, Shanghai Institutes for Biological Sciences, Chinese Academy of Sciences, University of Chinese Academy of Sciences, Shanghai 200031, P. R. China

Supplementary figures

Supplementary Fig. S1. Clustering of the ionome. The color scale is from saturated blue (significantly negative correlation) to white (no significant correlation) and then to saturated red (significantly positive correlation). (A) WT mice; (B) 3×Tg-AD mice; (C) Se-treated mice.

Supplementary Fig. S2. The decision-tree analysis for group classification. The total number of samples is 66. The text at the top of a branch node indicates the rules. The numbers shown in a tree leaf node represent the correctly classified and covered samples by the corresponding decision rules. The bottom of the branch node shows the branch condition: left for "yes" and right for ''no''. For example, if Fe >= 22 covers 38 samples with 30 correctly classified and 8 misclassified samples. The accuracy rate for the rule Fe >= 22 is 88% (58/66), and for the rule Se < 49 is 92% (61/66).

Supplementary Fig. S3. The elemental correlation network. Each node represents different elements. Differentially changed elements (DCEs) are highlighted in red (increased) and green (decreased). Green and red lines represent negative and positive correlation, respectively. Different color scales represent the magnitude of SCCs.

Supplementary Figure 1

A

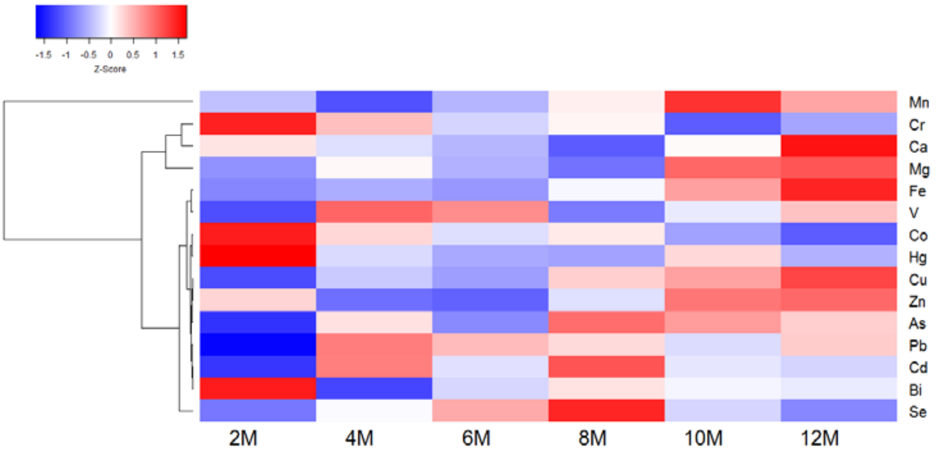

B

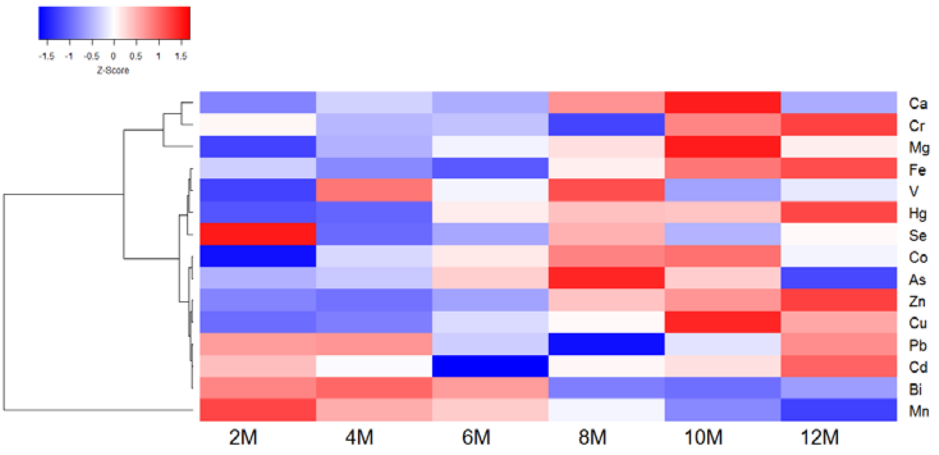

C

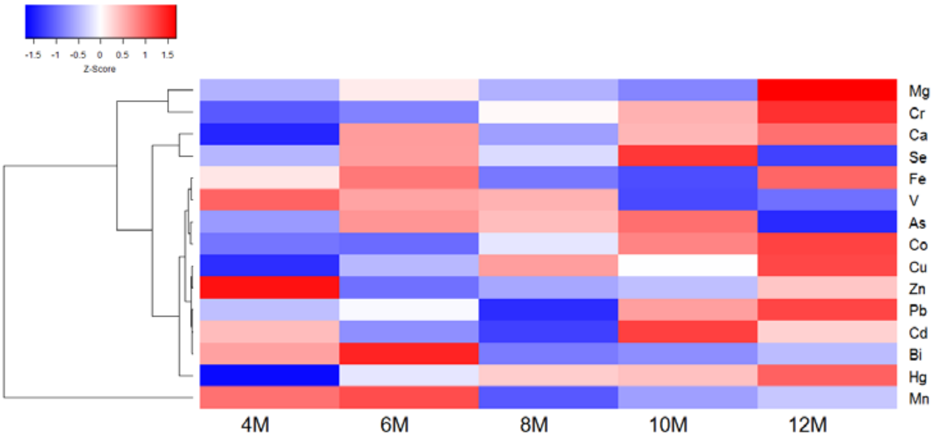

Supplementary Figure 2

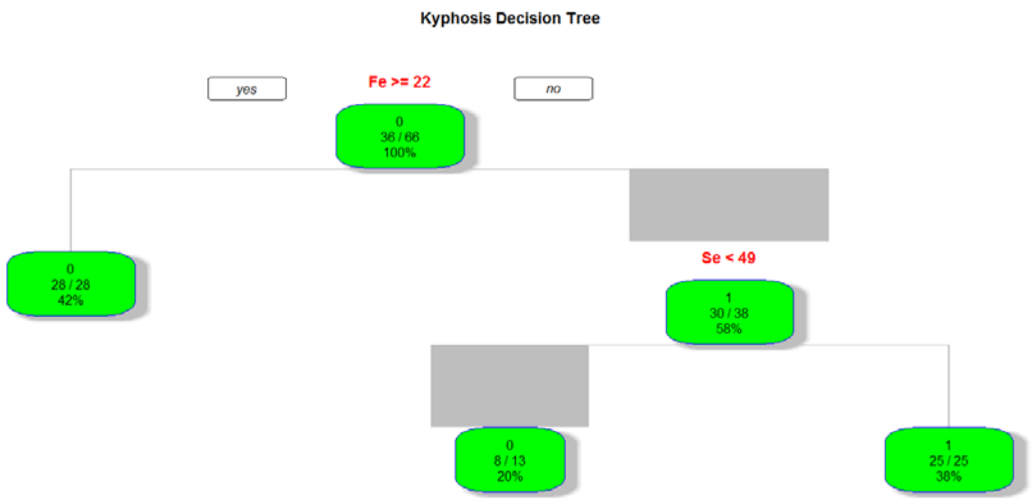

Supplementary Figure 3

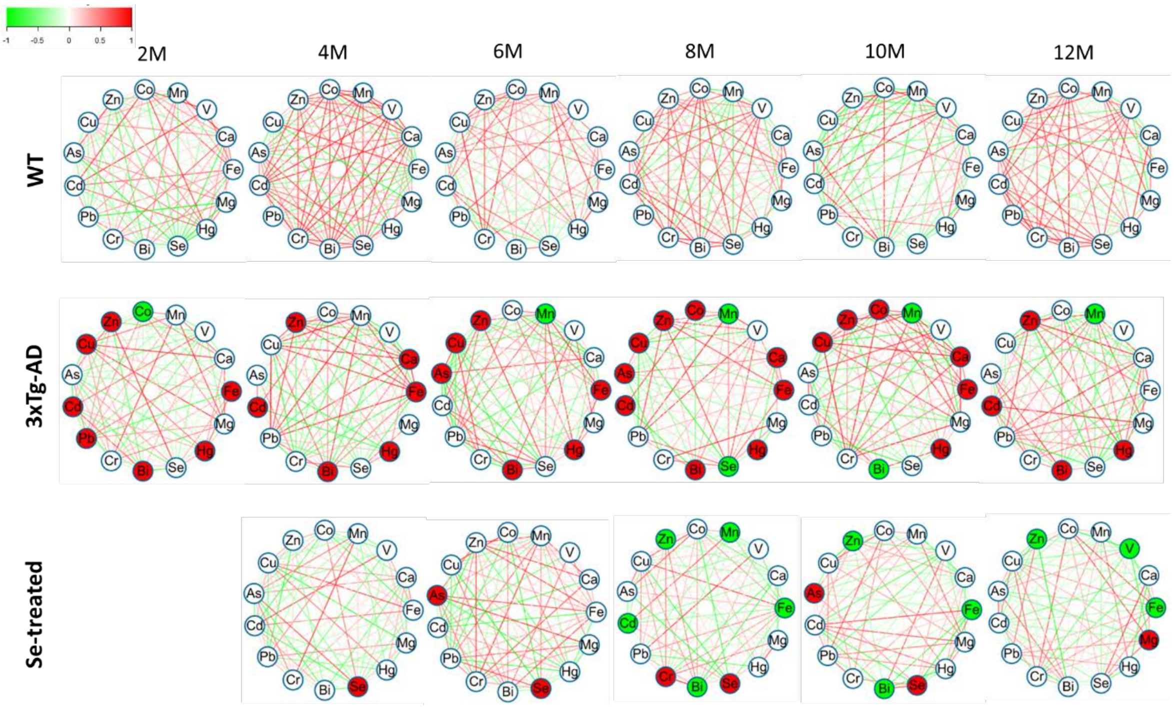

Supplementary tables

Supplementary Table 1. Distribution of pairwise SCCs in different groups

**Element-element interaction**	**WT group**						**3×Tg-AD group**						**Se-treated group**				
	**2-month**	**4-month**	**6-month**	**8-month**	**10-month**	**12-month**	**2-month**	**4-month**	**6-month**	**8-month**	**10-month**	**12-month**	**4-month**	**6-month**	**8-month**	**10-month**	**12-month**
Fe-Mg	0.086	-0.714	0.086	0.657	0.143	0.486	-0.086	-0.200	0.314	-0.086	0.486	0.086	-0.714	0.714	0.371	0.714	**0.943**
Fe-Ca	0.657	-0.657	0.314	**0.812**	**-0.841**	0.086	-0.200	**0.886**	-0.600	0.543	0.600	**0.771**	0.600	0.314	-0.314	-0.200	0.257
Fe-V	**0.771**	**-0.771**	-0.086	-0.200	0.257	-0.657	0.086	-0.657	0.200	0.486	-0.086	-0.257	0.429	0.486	0.371	-0.429	-0.714
Fe-Mn	0.543	-0.600	0.543	0.371	-0.200	**0.771**	-0.086	**0.886**	**0.886**	0.200	**0.829**	0.314	-0.319	0.371	-0.600	0.143	0.200
Fe-Co	**0.829**	-0.600	0.429	0.143	0.143	-0.143	-0.429	0.600	0.543	-0.143	0.657	-0.600	**-0.771**	0.257	0.486	-0.029	**0.829**
Fe-Zn	-0.086	-0.029	0.429	**0.771**	0.086	0.371	-0.029	**0.829**	0.371	**0.771**	0.657	0.657	0.200	**0.771**	-0.086	0.143	0.086
Fe-Cu	0.086	**0.829**	0.657	0.657	0.543	**0.829**	0.257	**0.943**	-0.029	-0.543	0.486	-0.200	**0.829**	0.371	**0.886**	0.143	0.486
Fe-As	0.200	-0.600	-0.086	0.314	0.086	-0.257	-0.429	0.086	-0.257	0.314	-0.086	-0.143	-0.143	**0.771**	0.314	-0.371	-0.486
Fe-Cd	**0.771**	-0.600	-0.086	0.314	0.314	-0.086	0.086	0.086	0.029	0.143	-0.143	-0.029	0.600	**-0.771**	-0.486	**0.829**	-0.371
Fe-Pb	0.543	-0.657	-0.086	-0.314	-0.314	-0.600	**-0.771**	**0.943**	0.257	0.314	0.200	0.714	-0.257	0.314	0.543	0.200	0.314
Fe-Cr	0.314	-0.657	0.600	0.314	0.029	-0.257	0.314	**-0.771**	-0.029	-0.086	0.600	0.371	0.143	0.429	0.486	-0.257	0.486
Fe-Bi	0.600	-0.657	-0.371	0.314	0.314	-0.086	0.086	0.600	-0.029	-0.029	0.143	-0.371	-0.314	**0.771**	0.493	**-0.886**	0.029
Fe-Se	-0.543	-0.714	-0.429	-0.086	-0.486	-0.257	0.371	-0.714	0.257	0.657	-0.429	**-0.829**	-0.486	-0.257	-0.600	**-0.943**	0.600
Fe-Hg	0.600	-0.200	0.371	0.486	-0.486	0.086	0.657	0.029	**0.829**	-0.143	0.200	0.714	0.200	-0.714	0.200	0.657	0.029
Mg-Ca	0.486	0.486	0.543	**0.812**	0.116	0.600	**0.886**	-0.086	0.314	0.429	**0.943**	0.543	-0.543	0.086	-0.600	-0.600	0.371
Mg-V	0.029	0.429	-0.086	-0.714	0.257	-0.714	-0.543	-0.314	**0.771**	0.029	**-0.771**	-0.371	0.257	0.029	-0.029	-0.429	**-0.771**
Mg-Mn	0.257	0.257	**0.829**	0.543	0.314	**0.829**	**0.886**	0.257	0.257	-0.257	**0.771**	**0.771**	0.116	0.543	-0.086	0.714	-0.029
Mg-Co	0.314	0.314	0.086	-0.486	-0.200	-0.257	0.543	0.143	-0.143	0.029	**0.829**	0.314	0.257	-0.086	0.143	0.486	**0.771**
Mg-Zn	0.600	-0.200	0.657	0.257	0.086	0.543	-0.200	-0.429	-0.371	-0.029	0.543	0.429	-0.429	0.600	-0.257	-0.143	0.371
Mg-Cu	0.486	**-0.886**	0.371	0.257	0.029	0.714	**0.886**	-0.029	-0.371	-0.086	**1.000**	-0.086	**-0.943**	-0.029	0.086	0.429	0.314

Mg-As	0.600	0.314	0.371	-0.143	-0.086	-0.371	-0.143	0.143	0.086	-0.429	-0.600	0.086	-0.029	**0.886**	-0.314	0.029	-0.600
Mg-Cd	0.029	0.314	0.029	-0.257	-0.029	-0.029	-0.086	0.143	-0.143	0.143	-0.657	**0.886**	0.029	-0.314	0.143	**0.771**	-0.429
Mg-Pb	**-0.771**	0.200	-0.371	-0.086	0.371	-0.200	0.429	-0.029	-0.086	-0.143	-0.086	0.486	0.543	0.657	-0.257	0.600	0.486
Mg-Cr	0.600	0.486	0.086	-0.257	0.371	-0.086	0.600	0.314	-0.543	**0.886**	**0.943**	0.600	-0.429	0.429	0.600	0.086	0.314
Mg-Bi	-0.486	0.486	0.143	-0.257	-0.029	-0.029	0.143	0.657	0.406	-0.200	-0.029	0.429	0.371	0.600	0.522	-0.657	-0.257
Mg-Se	**-0.771**	0.714	-0.143	-0.657	**-0.829**	-0.429	-0.257	0.600	-0.086	0.429	-0.086	-0.371	0.257	0.086	0.200	-0.543	0.657
Mg-Hg	0.657	0.029	**0.943**	-0.029	0.714	0.600	0.257	0.086	0.600	-0.657	0.257	0.029	-0.657	-0.714	**0.829**	0.543	-0.086
Ca-V	0.257	**0.886**	-0.086	-0.493	-0.580	-0.543	-0.429	**-0.829**	0.200	0.086	**-0.829**	-0.143	-0.086	0.143	-0.143	-0.086	0.086
Ca-Mn	**0.943**	**0.943**	0.600	0.232	0.696	0.429	0.600	**0.886**	**-0.771**	-0.257	**0.829**	**0.771**	-0.029	**0.771**	-0.314	**-0.829**	-0.486
Ca-Co	0.486	**0.943**	-0.086	-0.029	-0.522	-0.143	0.600	0.429	**-0.829**	0.086	**0.943**	-0.543	-0.486	0.143	-0.600	-0.543	-0.086
Ca-Zn	-0.257	0.714	0.257	0.464	0.406	0.257	-0.029	**0.886**	-0.714	0.543	0.429	**0.943**	-0.086	**0.771**	-0.371	0.486	0.143
Ca-Cu	0.543	-0.429	-0.086	0.319	-0.087	0.486	**0.829**	**0.943**	0.314	0.029	**0.943**	-0.543	0.429	-0.086	-0.371	-0.371	-0.314
Ca-As	-0.029	0.486	0.600	-0.058	-0.464	-0.714	-0.257	-0.257	**0.771**	0.543	-0.657	-0.257	0.486	0.086	-0.029	-0.200	-0.086
Ca-Cd	0.257	**0.943**	-0.086	0.203	-0.667	-0.086	0.086	-0.257	-0.600	0.257	-0.600	0.429	-0.029	**-0.771**	-0.429	-0.371	-0.600
Ca-Pb	-0.029	0.257	-0.371	-0.348	0.638	-0.029	0.600	**0.943**	**-0.771**	-0.029	-0.257	**0.943**	0.143	0.086	-0.143	-0.200	0.029
Ca-Cr	0.714	**1.000**	-0.257	0.203	0.029	-0.143	0.257	**-0.771**	0.029	0.086	**1.000**	0.314	0.314	**0.771**	-0.657	0.371	-0.657
Ca-Bi	0.429	**1.000**	0.200	0.203	-0.667	-0.086	0.029	0.600	-0.087	-0.257	0.086	-0.257	-0.714	-0.029	**-0.841**	-0.029	-0.086
Ca-Se	-0.657	**0.829**	0.086	-0.174	0.290	-0.371	-0.143	-0.371	-0.657	0.371	0.029	**-0.943**	0.257	0.143	0.029	0.257	**0.886**
Ca-Hg	0.486	0.371	0.486	0.377	0.667	**0.886**	0.429	0.486	-0.543	**-0.829**	0.371	0.714	0.371	-0.086	-0.314	0.314	**-0.771**
V-Mn	0.086	**0.829**	0.086	-0.657	**-0.771**	-0.600	-0.543	**-0.829**	0.371	**0.829**	-0.429	-0.257	-0.551	0.029	-0.429	-0.086	0.086
V-Co	**0.943**	**0.943**	0.029	**0.771**	**0.829**	0.657	0.029	-0.371	-0.257	-0.029	**-0.771**	-0.143	-0.543	0.086	-0.257	-0.086	**-0.886**
V-Zn	0.200	0.600	0.371	0.429	**-0.771**	-0.029	-0.600	-0.714	-0.029	**0.829**	0.029	-0.086	-0.029	0.257	0.086	-0.600	-0.314
V-Cu	0.257	-0.429	0.086	-0.314	-0.657	-0.714	-0.314	-0.714	-0.600	**-0.943**	**-0.771**	-0.314	0.029	0.200	0.200	0.257	-0.486
V-As	0.200	0.486	0.543	**0.771**	**0.886**	**0.771**	-0.543	-0.029	-0.257	-0.314	**0.829**	-0.600	-0.429	-0.086	-0.486	-0.429	0.371
V-Cd	**1.000**	**0.943**	0.200	0.714	**0.943**	0.600	0.657	0.143	0.314	-0.143	0.600	-0.086	**0.829**	-0.600	0.143	**-0.771**	-0.143
V-Pb	0.543	0.257	**0.829**	0.314	-0.600	**0.771**	-0.543	-0.714	0.257	**0.943**	0.543	0.086	-0.029	0.143	**0.886**	-0.657	-0.257

V-Cr	0.086	**0.886**	0.143	0.714	0.086	0.714	-0.600	0.314	**-0.886**	0.314	**-0.829**	-0.486	-0.600	-0.086	0.143	-0.314	-0.657
V-Bi	0.143	**0.886**	0.429	0.714	**0.943**	0.600	0.314	-0.714	**0.812**	0.543	-0.086	-0.600	0.086	0.600	0.319	**0.771**	0.143
V-Se	-0.257	0.714	-0.314	**0.829**	-0.543	**0.829**	-0.429	-0.029	0.371	**0.829**	-0.371	0.257	-0.371	0.086	0.143	0.314	-0.314
V-Hg	0.714	0.657	-0.029	-0.029	0.029	-0.257	-0.143	-0.486	0.371	-0.143	-0.429	-0.029	-0.429	-0.371	-0.543	**-0.771**	0.029
Mn-Co	0.257	**0.886**	0.086	**-0.771**	**-0.771**	-0.086	0.543	0.600	0.600	0.371	0.714	-0.314	0.232	-0.314	0.314	0.429	0.314
Mn-Zn	-0.486	**0.771**	0.714	-0.086	**0.886**	0.600	-0.257	0.657	0.600	0.600	**0.771**	**0.829**	0.000	**0.829**	0.029	-0.200	-0.200
Mn-Cu	0.486	-0.257	0.543	0.657	0.600	**0.943**	0.657	**0.943**	-0.429	**-0.771**	**0.771**	-0.657	-0.348	-0.486	-0.314	0.200	0.029
Mn-As	-0.257	0.429	0.486	-0.257	**-0.771**	-0.143	0.143	0.029	-0.657	-0.371	-0.143	0.200	0.609	0.429	0.086	0.486	-0.371
Mn-Cd	0.086	**0.886**	-0.200	-0.714	**-0.829**	0.143	-0.371	0.029	0.486	0.143	-0.200	**0.771**	-0.348	-0.543	**0.771**	0.371	-0.257
Mn-Pb	0.086	0.429	-0.086	-0.486	0.714	-0.143	0.314	**0.943**	0.657	**0.943**	0.143	0.600	0.522	0.657	-0.429	0.314	-0.200
Mn-Cr	0.543	**0.943**	0.257	-0.714	0.029	0.086	**0.829**	-0.657	-0.371	0.086	**0.829**	0.429	**0.841**	**0.886**	-0.371	-0.086	0.543
Mn-Bi	0.600	**0.943**	-0.143	-0.714	**-0.829**	0.143	0.029	**0.886**	0.348	0.314	0.314	0.314	-0.029	0.029	-0.174	-0.029	0.257
Mn-Se	-0.486	0.657	-0.543	**-0.829**	-0.029	-0.086	-0.257	-0.371	0.657	0.543	-0.314	-0.600	0.174	0.543	0.029	-0.029	-0.429
Mn-Hg	0.257	0.257	**0.943**	-0.143	0.486	0.543	-0.086	0.200	**0.829**	0.257	0.371	0.600	-0.464	-0.486	0.086	-0.029	0.600
Co-Zn	0.257	**0.771**	0.657	0.543	-0.600	0.657	-0.257	0.257	0.200	0.086	0.257	-0.714	0.429	-0.029	0.200	-0.714	0.143
Co-Cu	0.371	-0.314	**0.771**	-0.086	-0.657	-0.257	0.314	0.486	-0.143	0.086	**0.829**	**0.771**	-0.429	**0.943**	**0.771**	**0.829**	0.429
Co-As	0.314	0.543	-0.543	0.543	**0.886**	0.429	0.029	0.200	-0.600	-0.029	**-0.771**	0.086	0.314	-0.086	**0.771**	-0.029	-0.486
Co-Cd	**0.943**	**1.000**	**0.829**	**0.943**	**0.943**	**0.943**	-0.143	0.714	0.314	**0.886**	-0.657	0.257	**-0.829**	-0.371	-0.029	0.143	-0.086
Co-Pb	0.314	0.143	-0.314	0.200	**-0.886**	0.600	0.543	0.486	0.600	0.200	-0.371	-0.429	-0.314	**-0.771**	0.086	**0.771**	0.029
Co-Cr	0.371	**0.943**	**0.886**	**0.943**	-0.371	**0.829**	0.257	-0.486	0.029	-0.029	**0.943**	0.257	-0.029	-0.314	0.257	0.486	**0.771**
Co-Bi	0.086	**0.943**	0.429	**0.943**	**0.943**	**0.943**	-0.314	0.543	0.029	-0.657	-0.029	0.429	-0.086	0.486	0.406	-0.029	0.086
Co-Se	-0.486	**0.771**	0.314	**0.943**	-0.143	0.714	-0.257	-0.371	0.257	0.029	0.086	0.600	0.600	**-0.886**	**-0.771**	0.086	0.314
Co-Hg	**0.829**	0.600	0.200	0.543	-0.257	0.257	-0.200	-0.257	0.600	0.086	0.314	**-0.886**	0.086	0.486	0.257	-0.029	0.029
Zn-Cu	-0.029	0.314	**0.829**	0.257	**0.771**	0.371	-0.314	**0.771**	-0.371	-0.714	0.543	**-0.771**	0.371	-0.086	0.143	**-0.886**	-0.543
Zn-As	**0.886**	0.086	0.143	**0.829**	**-0.771**	0.086	0.600	-0.086	-0.600	0.200	0.257	-0.143	0.257	0.657	0.257	0.486	**-0.771**
Zn-Cd	0.200	**0.771**	0.486	0.714	-0.714	**0.771**	-0.600	-0.257	0.657	0.086	0.086	0.371	-0.257	**-0.829**	-0.086	0.314	-0.429

Zn-Pb	-0.486	-0.143	-0.029	0.029	0.371	0.257	0.429	**0.771**	0.600	**0.771**	0.714	**0.829**	**-0.771**	0.486	0.257	-0.143	0.600
Zn-Cr	-0.143	0.714	0.714	0.714	-0.314	0.600	0.029	-0.600	-0.086	0.029	0.429	0.143	0.143	**0.886**	0.543	-0.086	-0.200
Zn-Bi	-0.657	0.714	0.371	0.714	-0.714	**0.771**	-0.714	0.257	0.116	0.257	0.314	-0.200	-0.486	0.314	0.609	-0.371	**-0.943**
Zn-Se	-0.486	0.314	-0.200	0.371	0.086	0.257	**0.886**	-0.486	**0.829**	**0.771**	**-0.771**	**-0.886**	0.143	0.143	0.143	-0.086	0.029
Zn-Hg	0.600	0.486	**0.771**	0.257	0.086	0.600	0.200	0.371	0.200	-0.371	0.257	**0.829**	0.086	-0.657	-0.429	0.486	-0.029
Cu-As	-0.257	-0.657	-0.314	-0.086	-0.714	-0.314	-0.543	-0.143	**0.771**	0.486	-0.600	0.029	-0.257	0.029	0.657	-0.543	0.371
Cu-Cd	0.257	-0.314	0.371	-0.143	-0.600	-0.086	0.257	-0.143	**-0.829**	0.086	-0.657	-0.257	0.257	-0.314	-0.429	-0.029	0.543
Cu-Pb	-0.257	-0.486	-0.086	-0.543	0.371	-0.314	0.086	**1.000**	**-0.771**	**-0.829**	-0.086	-0.429	-0.600	-0.714	0.543	0.486	0.086
Cu-Cr	0.486	-0.429	**0.943**	-0.143	0.029	-0.143	0.429	**-0.829**	**0.829**	-0.429	**0.943**	0.200	0.200	-0.429	0.371	0.371	0.657
Cu-Bi	-0.257	-0.429	-0.029	-0.143	-0.600	-0.086	0.371	0.714	**-0.812**	-0.429	-0.029	0.143	-0.200	0.600	0.464	-0.029	0.486
Cu-Se	-0.200	**-0.771**	-0.314	-0.257	-0.029	-0.200	-0.257	-0.543	**-0.771**	**-0.886**	-0.086	0.429	-0.486	**-0.943**	**-0.829**	-0.086	0.086
Cu-Hg	0.486	0.086	0.600	0.600	-0.257	0.486	0.600	0.257	-0.429	-0.029	0.257	**-0.771**	0.600	0.314	0.029	-0.029	0.314
As-Cd	0.200	0.543	-0.371	0.657	**0.943**	0.543	**-0.886**	0.657	**-0.943**	0.029	**0.771**	0.200	-0.600	-0.314	-0.486	0.143	0.714
As-Pb	-0.371	0.486	0.429	0.257	-0.600	0.657	0.486	-0.143	**-1.000**	-0.257	0.600	-0.543	0.257	0.543	-0.029	0.200	-0.486
As-Cr	-0.029	0.486	-0.486	0.657	0.086	0.714	0.314	0.486	0.600	**-0.771**	-0.657	0.486	0.543	0.543	0.029	0.086	-0.143
As-Bi	-0.314	0.486	0.143	0.657	**0.943**	0.543	-0.657	0.143	-0.667	-0.143	0.257	**0.886**	**-0.771**	0.429	0.029	0.143	0.657
As-Se	-0.714	**0.771**	-0.371	0.486	-0.314	**0.829**	0.429	-0.257	**-0.886**	-0.371	-0.371	0.314	**0.771**	-0.086	**-0.829**	0.429	-0.086
As-Hg	0.600	0.029	0.314	-0.143	-0.029	-0.371	-0.600	**-0.771**	-0.429	-0.257	-0.200	0.143	-0.143	**-0.771**	0.029	-0.029	-0.200
Cd-Pb	0.543	0.143	-0.257	0.143	**-0.771**	0.714	-0.371	-0.143	**0.943**	-0.029	0.543	0.371	0.143	-0.086	-0.086	0.486	-0.257
Cd-Cr	0.086	**0.943**	0.543	**1.000**	-0.086	**0.943**	-0.657	0.143	-0.657	0.029	-0.600	0.657	-0.257	-0.543	-0.257	-0.143	0.371
Cd-Bi	0.143	**0.943**	**0.829**	**1.000**	**1.000**	**1.000**	**0.771**	0.143	0.725	**-0.886**	0.714	0.486	0.371	-0.543	0.000	**-0.886**	0.371
Cd-Se	-0.257	**0.771**	0.657	**0.886**	-0.371	**0.771**	-0.600	-0.257	**0.943**	0.143	-0.429	-0.143	**-0.771**	0.143	0.371	**-0.771**	-0.429
Cd-Hg	0.714	0.600	-0.029	0.429	-0.200	0.371	0.314	**-0.771**	0.200	0.086	0.371	0.143	-0.371	0.371	-0.029	0.657	0.143
Pb-Cr	-0.371	0.257	-0.029	0.143	0.657	**0.886**	0.029	**-0.829**	-0.600	0.143	-0.257	0.200	0.257	0.543	0.143	0.714	-0.086
Pb-Bi	0.714	0.257	-0.086	0.143	**-0.771**	0.714	-0.429	0.714	0.667	0.486	0.371	-0.486	0.200	0.086	0.348	-0.486	**-0.771**
Pb-Se	0.314	0.257	-0.600	0.086	-0.086	**0.829**	0.086	-0.543	**0.886**	0.657	**-0.943**	**-0.886**	0.086	**0.771**	-0.257	-0.029	0.029

Pb-Hg	-0.086	-0.429	-0.257	-0.257	0.600	0.314	-0.200	0.257	0.429	-0.029	0.200	0.543	-0.543	**-0.829**	-0.657	0.543	0.429
Cr-Bi	-0.086	**1.000**	0.086	**1.000**	-0.086	**0.943**	-0.257	-0.371	**-0.986**	0.029	0.086	0.543	-0.200	-0.143	**0.928**	-0.086	0.314
Cr-Se	-0.371	**0.829**	-0.143	**0.886**	-0.429	**0.886**	0.200	0.600	-0.600	0.600	0.029	-0.143	-0.029	0.371	0.143	0.486	-0.257
Cr-Hg	0.257	0.371	0.314	0.429	0.429	0.314	0.029	-0.257	-0.314	-0.371	0.371	0.200	-0.086	-0.543	0.314	0.429	0.486
Bi-Se	-0.086	**0.829**	0.657	**0.886**	-0.371	**0.771**	**-0.771**	-0.086	0.638	0.143	-0.429	0.371	-0.657	-0.371	0.058	**0.771**	0.086
Bi-Hg	-0.143	0.371	-0.029	0.429	-0.200	0.371	0.086	0.086	0.232	-0.086	**0.886**	-0.143	-0.200	-0.371	0.145	**-0.886**	-0.143
Se-Hg	**-0.771**	0.200	-0.371	0.371	-0.486	0.029	0.371	0.486	0.314	-0.314	-0.371	-0.657	0.086	-0.371	-0.029	-0.429	-0.714
**Number of significant SCCs**	12	34	11	19	24	22	10	23	24	17	27	19	10	20	11	15	11

Supplementary Table 2. Distribution of DCCs between different groups

Element-element interaction	3×Tg-AD Vs. WT (AD-related DCCs)						Se-treated Vs.3×Tg-AD (Se-related DCCs)				
	2-month	4-month	6-month	8-month	10-month	12-month	4-month	6-month	8-month	10-month	12-month
AD-specific (33)											
Fe-Ca	-0.857142857	**1.54285714**	-0.91428571	-0.26882231	**1.440668**	0.68571429	-0.28571429	0.91428571	-0.85714286	-0.8	-0.51428571
Fe-Bi	-0.514285714	**1.25714286**	0.34244002	-0.34285714	-0.1714286	-0.2857143	-0.91428571	0.80041712	0.521376809	-1.0285714	0.4
Mg-Pb	**1.2**	-0.2285714	0.285714286	-0.05714286	-0.4571429	0.68571429	0.571428571	0.74285714	-0.11428571	0.68571429	0
Mg-Cr	0	-0.1714286	-0.62857143	**1.142857143**	0.5714286	0.68571429	-0.74285714	0.97142857	-0.28571429	-0.8571429	-0.28571429
Mg-Se	0.514285714	-0.1142857	0.057142857	**1.085714286**	0.7428571	0.05714286	-0.34285714	0.17142857	-0.22857143	-0.4571429	1.028571429
Ca-V	-0.685714286	**-1.7142857**	0.285714286	0.578519666	-0.2488004	0.4	0.742857143	-0.05714286	-0.22857143	0.74285714	0.228571429
Ca-Cd	-0.171428571	**-1.2**	-0.51428571	0.054222995	0.0667367	0.51428571	0.228571429	-0.17142857	-0.68571429	0.22857143	-1.02857143
V-Co	-0.914285714	**-1.3142857**	-0.28571429	-0.8	**-1.6**	-0.8	-0.17142857	0.34285714	-0.22857143	0.68571429	-0.74285714
V-Zn	-0.8	**-1.3142857**	-0.4	0.4	0.8	-0.0571429	0.685714286	0.28571429	-0.74285714	-0.6285714	-0.22857143
V-Cr	-0.685714286	-0.5714286	-1.02857143	-0.4	-0.9142857	**-1.2**	-0.91428571	0.8	-0.17142857	0.51428571	-0.17142857
V-Bi	0.171428571	**-1.6**	0.383108021	-0.17142857	-1.0285714	**-1.2**	0.8	-0.21167945	-0.22398307	0.85714286	0.742857143
V-Hg	-0.857142857	**-1.1428571**	0.4	-0.11428571	-0.4571429	0.22857143	0.057142857	-0.74285714	-0.4	-0.3428571	0.057142857
Mn-Co	0.285714286	-0.2857143	0.514285714	**1.142857143**	**1.4857143**	-0.2285714	-0.36809159	-0.91428571	-0.05714286	-0.2857143	0.628571429
Mn-Bi	-0.571428571	-0.0571429	0.490719764	1.028571429	**1.1428571**	0.17142857	-0.91470284	-0.31929119	-0.48821702	-0.3428571	-0.05714286
Mn-Se	0.228571429	-1.0285714	**1.2**	**1.371428571**	-0.2857143	-0.5142857	0.545359882	-0.11428571	-0.51428571	0.28571429	0.171428571
Co-Zn	-0.514285714	-0.5142857	-0.45714286	-0.45714286	0.8571429	**-1.3714286**	0.171428571	-0.22857143	0.114285714	-0.9714286	0.857142857
Co-As	-0.285714286	-0.3428571	-0.05714286	-0.57142857	**-1.6571429**	-0.3428571	0.114285714	0.51428571	0.8	0.74285714	-0.57142857
Co-Cr	-0.114285714	**-1.4285714**	-0.85714286	-0.97142857	**1.3142857**	-0.5714286	0.457142857	-0.34285714	0.285714286	-0.4571429	0.514285714
Co-Hg	-1.028571429	-0.8571429	0.4	-0.45714286	0.5714286	**-1.1428571**	0.342857143	-0.11428571	0.171428571	-0.3428571	0.914285714
Zn-Cr	0.171428571	**-1.3142857**	-0.8	-0.68571429	0.7428571	-0.4571429	0.742857143	0.97142857	0.514285714	-0.5142857	-0.34285714
Zn-Se	**1.371428571**	-0.8	1.028571429	0.4	-0.8571429	**-1.1428571**	0.628571429	-0.68571429	-0.62857143	0.68571429	0.914285714
Cu-As	-0.285714286	0.51428571	**1.085714286**	0.571428571	0.1142857	0.34285714	-0.11428571	-0.74285714	0.171428571	0.05714286	0.342857143

Cu-Cd	0	0.17142857	**-1.2**	0.228571429	-0.0571429	-0.1714286	0.4	0.51428571	-0.51428571	0.62857143	0.8
As-Cr	0.342857143	0	**1.085714286**	**-1.42857143**	-0.7428571	-0.2285714	0.057142857	-0.05714286	0.8	0.74285714	-0.62857143
As-Se	**1.142857143**	-1.0285714	-0.51428571	-0.85714286	-0.0571429	-0.5142857	1.028571429	0.8	-0.45714286	0.8	-0.4
As-Hg	**-1.2**	-0.8	-0.74285714	-0.11428571	-0.1714286	0.51428571	0.628571429	-0.34285714	0.285714286	0.17142857	-0.34285714
Cd-Pb	-0.914285714	-0.2857143	**1.2**	-0.17142857	**1.3142857**	-0.3428571	0.285714286	-1.02857143	-0.05714286	-0.0571429	-0.62857143
Cd-Cr	-0.742857143	-0.8	**-1.2**	-0.97142857	-0.5142857	-0.2857143	-0.4	0.11428571	-0.28571429	0.45714286	-0.28571429
Cd-Hg	-0.4	**-1.3714286**	0.228571429	-0.34285714	0.5714286	-0.2285714	0.4	0.17142857	-0.11428571	0.28571429	0
Pb-Bi	**-1.142857143**	0.45714286	0.752450977	0.342857143	**1.1428571**	**-1.2**	-0.51428571	-0.58102241	-0.13785166	-0.8571429	-0.28571429
Pb-Se	-0.228571429	-0.8	**1.485714286**	0.571428571	-0.8571429	**-1.7142857**	0.628571429	-0.11428571	-0.91428571	0.91428571	0.914285714
Cr-Bi	-0.171428571	**-1.3714286**	**-1.07132505**	-0.97142857	0.1714286	-0.4	0.171428571	0.84275362	0.899062228	-0.1714286	-0.22857143
Se-Hg	**1.142857143**	0.28571429	0.685714286	-0.68571429	0.1142857	-0.6857143	-0.4	-0.68571429	0.285714286	-0.0571429	-0.05714286
Overlapped (30)											
Fe-Mn	-0.628571429	**1.48571429**	0.342857143	-0.17142857	1.0285714	-0.4571429	**-1.20458836**	-0.51428571	-0.8	-0.6857143	-0.11428571
Fe-Co	**-1.257142857**	**1.2**	0.114285714	-0.28571429	0.5142857	-0.4571429	**-1.37142857**	-0.28571429	0.628571429	-0.6857143	**1.428571429**
Fe-Cu	0.171428571	0.11428571	-0.68571429	**-1.2**	-0.0571429	-1.0285714	-0.11428571	0.4	**1.428571429**	-0.3428571	0.685714286
Fe-Pb	**-1.314285714**	**1.6**	0.342857143	0.628571429	0.5142857	**1.31428571**	**-1.2**	0.05714286	0.228571429	0	-0.4
Ca-Mn	-0.342857143	-0.0571429	**-1.37142857**	-0.48905127	0.1328462	0.34285714	-0.91470284	**1.54285714**	-0.05714286	**-1.6571429**	**-1.25714286**
Ca-Co	0.114285714	-0.5142857	-0.74285714	0.114702837	**1.4646511**	-0.4	-0.91428571	0.97142857	-0.68571429	**-1.4857143**	0.457142857
Ca-Cu	0.285714286	**1.37142857**	0.4	-0.29030264	1.0298228	-1.0285714	-0.51428571	-0.4	-0.4	**-1.3142857**	0.228571429
Ca-Cr	-0.457142857	**-1.7714286**	0.285714286	-0.11720558	0.9710114	0.45714286	**1.085714286**	0.74285714	-0.74285714	-0.6285714	-0.97142857
Ca-Se	0.514285714	**-1.2**	-0.74285714	0.545359882	-0.2613141	-0.5714286	0.628571429	0.8	-0.34285714	0.22857143	**1.828571429**
Ca-Hg	-0.057142857	0.11428571	-1.02857143	**-1.2054226**	-0.2953081	-0.1714286	-0.11428571	0.45714286	0.514285714	-0.0571429	**-1.48571429**
V-Mn	-0.628571429	**-1.6571429**	0.285714286	**1.485714286**	0.3428571	0.34285714	0.277788945	-0.34285714	**-1.25714286**	0.34285714	0.342857143
V-As	-0.742857143	-0.5142857	-0.8	**-1.08571429**	-0.0571429	**-1.3714286**	-0.4	0.17142857	-0.17142857	**-1.2571429**	0.971428571
V-Pb	**-1.085714286**	-0.9714286	-0.57142857	0.628571429	**1.1428571**	-0.6857143	0.685714286	-0.11428571	-0.05714286	**-1.2**	-0.34285714
Mn-Cu	0.171428571	**1.2**	-0.97142857	**-1.42857143**	0.1714286	**-1.6**	**-1.29071976**	-0.05714286	0.457142857	-0.5714286	0.685714286

Mn-As	0.4	-0.4	**-1.14285714**	-0.11428571	0.6285714	0.34285714	0.580188159	**1.08571429**	0.457142857	0.62857143	-0.57142857
Mn-Pb	0.228571429	0.51428571	0.742857143	**1.428571429**	-0.5714286	0.74285714	-0.42106321	0	**-1.37142857**	0.17142857	-0.8
Mn-Cr	0.285714286	**-1.6**	-0.62857143	0.8	0.8	0.34285714	**1.497810859**	**1.25714286**	-0.45714286	-0.9142857	0.114285714
Co-Cu	-0.057142857	0.8	-0.91428571	0.171428571	**1.4857143**	1.02857143	-0.91428571	**1.08571429**	0.685714286	0	-0.34285714
Co-Cd	**-1.085714286**	-0.2857143	-0.51428571	-0.05714286	**-1.6**	-0.6857143	**-1.54285714**	-0.68571429	-0.91428571	0.8	-0.34285714
Co-Bi	-0.4	-0.4	-0.39958288	**-1.6**	-0.9714286	-0.5142857	-0.62857143	0.45672573	**1.062982582**	0	-0.34285714
Co-Se	0.228571429	**-1.1428571**	-0.05714286	-0.91428571	0.2285714	-0.1142857	0.971428571	**-1.14285714**	-0.8	0	-0.28571429
Zn-Cu	-0.285714286	0.45714286	**-1.2**	-0.97142857	-0.2285714	**-1.1428571**	-0.4	0.28571429	0.857142857	**-1.4285714**	0.228571429
Cu-Pb	0.342857143	**1.48571429**	-0.68571429	-0.28571429	-0.4571429	-0.1142857	**-1.6**	0.05714286	**1.371428571**	0.57142857	0.514285714
Cu-Bi	0.628571429	**1.14285714**	-0.78310802	-0.28571429	0.5714286	0.22857143	-0.91428571	**1.41167945**	0.892388257	0	0.342857143
Cu-Hg	0.114285714	0.17142857	-1.02857143	-0.62857143	0.5142857	**-1.2571429**	0.342857143	0.74285714	0.057142857	-0.2857143	**1.085714286**
As-Cd	**-1.085714286**	0.11428571	-0.57142857	-0.62857143	-0.1714286	-0.3428571	**-1.25714286**	0.62857143	-0.51428571	-0.6285714	0.514285714
As-Pb	0.857142857	-0.6285714	**-1.42857143**	-0.51428571	**1.2**	**-1.2**	0.4	**1.54285714**	0.228571429	-0.4	0.057142857
Cd-Bi	0.628571429	-0.8	-0.10385763	**-1.88571429**	-0.2857143	-0.5142857	0.228571429	**-1.26757094**	0.885714286	**-1.6**	-0.11428571
Pb-Cr	0.4	**-1.0857143**	-0.57142857	0	-0.9142857	-0.6857143	**1.085714286**	**1.14285714**	0	0.97142857	-0.28571429
Bi-Hg	0.228571429	-0.2857143	0.260479843	-0.51428571	**1.0857143**	-0.5142857	-0.28571429	-0.60333699	0.230657045	**-1.7714286**	0
Se-specific (19)											
Fe-V	-0.685714286	0.11428571	0.285714286	0.685714286	-0.3428571	0.4	**1.085714286**	0.28571429	-0.11428571	-0.3428571	-0.45714286
Fe-Se	0.914285714	0	0.685714286	0.742857143	0.0571429	-0.5714286	0.228571429	-0.51428571	**-1.25714286**	-0.5142857	**1.428571429**
Fe-Hg	0.057142857	0.22857143	0.457142857	-0.62857143	0.6857143	0.62857143	0.171428571	**-1.54285714**	0.342857143	0.45714286	-0.68571429
Mg-Ca	0.4	-0.5714286	-0.22857143	-0.38310802	0.8269029	-0.0571429	-0.45714286	-0.22857143	-1.02857143	**-1.5428571**	-0.17142857
Mg-Cd	-0.114285714	-0.1714286	-0.17142857	0.4	-0.6285714	0.91428571	-0.11428571	-0.17142857	0	**1.42857143**	**-1.31428571**
Mg-Hg	-0.4	0.05714286	-0.34285714	-0.62857143	-0.4571429	-0.5714286	-0.74285714	**-1.31428571**	**1.485714286**	0.28571429	-0.11428571
Ca-Zn	0.228571429	0.17142857	-0.97142857	0.079040314	0.0227317	0.68571429	-0.97142857	**1.48571429**	-0.91428571	0.05714286	-0.8
Ca-Bi	-0.4	-0.4	-0.28696566	-0.46006272	0.752451	-0.1714286	**-1.31428571**	0.05839423	-0.58352514	-0.1142857	0.171428571
V-Cu	-0.571428571	-0.2857143	-0.68571429	-0.62857143	-0.1142857	0.4	0.742857143	0.8	**1.142857143**	1.02857143	-0.17142857

V-Cd	-0.342857143	-0.8	0.114285714	-0.85714286	-0.3428571	-0.6857143	0.685714286	-0.91428571	0.285714286	**-1.3714286**	-0.05714286
Mn-Hg	-0.342857143	-0.0571429	-0.11428571	0.4	-0.1142857	0.05714286	-0.66381683	**-1.31428571**	-0.17142857	-0.4	0
Co-Pb	0.228571429	0.34285714	0.914285714	0	0.5142857	-1.0285714	-0.8	**-1.37142857**	-0.11428571	**1.14285714**	0.457142857
Zn-As	-0.285714286	-0.1714286	-0.74285714	-0.62857143	1.0285714	-0.2285714	0.342857143	**1.25714286**	0.057142857	0.22857143	-0.62857143
Zn-Cd	-0.8	-1.0285714	0.171428571	-0.62857143	0.8	-0.4	0	**-1.48571429**	-0.17142857	0.22857143	-0.8
Zn-Pb	0.914285714	0.91428571	0.628571429	0.742857143	0.3428571	0.57142857	**-1.54285714**	-0.11428571	-0.51428571	-0.8571429	-0.22857143
Cu-Cr	-0.057142857	-0.4	-0.11428571	-0.28571429	0.9142857	0.34285714	1.028571429	**-1.25714286**	0.8	-0.5714286	0.457142857
As-Bi	-0.342857143	-0.3428571	-0.80959383	-0.8	-0.6857143	0.34285714	-0.91428571	**1.09530812**	0.171845695	-0.1142857	-0.22857143
Pb-Hg	-0.114285714	0.68571429	0.685714286	0.228571429	-0.4	0.22857143	-0.8	**-1.25714286**	-0.62857143	0.34285714	-0.11428571
Bi-Se	-0.685714286	-0.9142857	-0.01939472	-0.74285714	-0.0571429	-0.4	-0.57142857	-1.00917671	-0.08488004	**1.2**	-0.28571429

Supplementary Table 3. The list of differentially changed elements (DCEs) between Se-treated and WT mice

Age	DCEs with increased level	DCEs with decreased level
4-month	Mg, Zn, Cu, As, Bi, Se, Hg	Cr
6-month	Ca, Zn, Cu, As, Bi, Se, Hg	-
8-month	Ca, Zn, Cu, As, Hg	Mn, Pb, Bi,
10-month	Ca, Zn, As, Hg, Pb	V, Mn
12-month	Ca, Zn, Cd, Pb, Cr, Hg	Fe, V, Mn
